# Endodontic Treatment in Artificial Deciduous Teeth by Manual and Mechanical Instrumentation: A Pilot Study

**DOI:** 10.5005/jp-journals-10005-1566

**Published:** 2018

**Authors:** Fernanda Hecksher, Bruno Vidigal, Patricia Coelho, Diassanam Otoni, Christiano Alvarenga, Eduardo Nunes

**Affiliations:** 1-3,6 Department of Dentistry, PUC-Minas, Minas Gerais, Brazil; 1,4,5 Department of Dentistry, São Leopoldo Mandic, Minas Gerais, Brazil; 3 Department of Dentistry, Fainor, Minas Gerais, Brazil; 4 Department of Dentistry, Newton Paiva, Minas Gerais, Brazil

**Keywords:** Deciduous teeth, Endodontics, Instrumentation

## Abstract

**Introduction:**

“*In vitro*” studies seek to simulate actual biological conditions in the laboratory and providing principles to be studied later, to facilitate the development of new techniques of root instrumentation in primary teeth and to ensure the integrity and function of the element.

**Aim:**

This study aimed to demonstrate the technological advances in endodontics by endodontic treatment performed on artificial primary teeth using a rotary instrumentation system and reciprocation.

**Materials and methods:**

Instrumentation of the root canal was performed via a manual, rotatory and Reciproc system.

**Results:**

The rotatory systems can facilitate endodontic treatment in one session.

**Conclusion:**

These procedures become increasingly easy and rapid with the help of technological advances in dentistry.

**How to cite this article:**

Hecksher F, Vidigal B, Coelho P, Otoni D, Alvarenga C, Nunes E. Endodontic Treatment in Artificial Deciduous Teeth by Manual and Mechanical Instrumentation: A Pilot Study. Int J Clin Pediatr Dent, 2018;11(6):510-512

## INTRODUCTION

The dental pulp of deciduous teeth can become involved much earlier than permanent teeth in advance of carious lesions. In addition, exposure of the pulp can also occur much more often during cavity preparation due to the thickness of the enamel and dentin, which are very often thinner in deciduous teeth. It is also noteworthy that traumatic injuries, especially in anterior teeth, occur frequently, contributing to a public health problem.^1,2^

The main goal of dentistry is to maintain the integrity and function of the primary dentition to its physiological exfoliation. When the deciduous pulp is compromised, endodontic treatment should be performed to preserve the integrity and function of the tooth and its supporting tissues.^3^ The instrumentation of the root canal may be performed with manual or mechanical tools.^4^

The instruments that incorporate reciprocating movements were introduced in the market to model the dental channel using only a file. These file instruments have a different mechanism compared with other previously developed files. The system is designed to be used with a reciprocating.^5^

Endodontic treatment with rotary systems can contribute significantly to reduce the duration of clinical care for pediatric patients. However, it is essential to evaluate the dentin wear created by both hands and by rotary instruments to analyze the safety of this procedure in the different root thirds and the presence of resorption.^6^ This study aims to demonstrate the potential applications of technological advances in endodontics in pediatric dentistry.

## METHODOLOGY

The sample comprised artificial deciduous teeth with coronary and root pulp (Denarte, São Paulo, SP, Brazil) divided into three groups: group one was instrumented with a manual technique (G1), group 2 used a roundabout technique (G2) and group 3 used a reciprocating instrumented technique (G3).

The manual instrumentation group used instrumentation techniques such as the Scheduling Crown-Down Manual, which were performed with Kerr type files 1st series 21 mm (VDW, Munich, Germany). The instrumentation system used RECIPROC file Reciproc 25/0.08 (VDW, Munich, Germany), while the instrumentation group's rotary system utilized Mtwo files of sizes 10/0.4, 15/0.5, 20/0.6, 25/0.6–21 mm, adapted to the VDW engine (VDW, Munich, Germany).

The procedure to gain access to the root canal was performed by an endodontist experienced in crown-opening with a round burn° 1011, exploitation of channels C file–Pilot # 10 0.2, 21 mm, to the working length (CT) without resistance, and widening the entrance of the conduits with Gates Glidden drills using #2–4. It was then performed at timed instrumentation conduits. We used a stopwatch to measure the instrumentation time in each root canal.

## RESULTS

**Graph 1:**
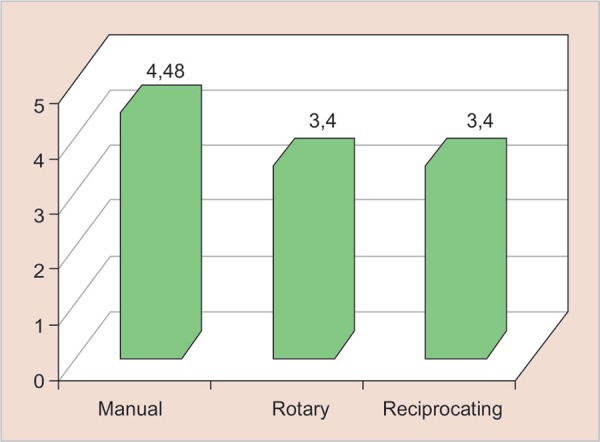
Required time for instrumentation for the three systems

The comparisons between the manual instrumentation group and the rotary and reciprocating systems showed that all the three groups were able to perform the instrumentation of the conduits. The average time required for the manual technique was 4.4 minutes, while with the rotary and reciprocating systems required 3.4 minutes (Graph 1).

## CLINICAL APPLICATIONS

**Graph 2:**
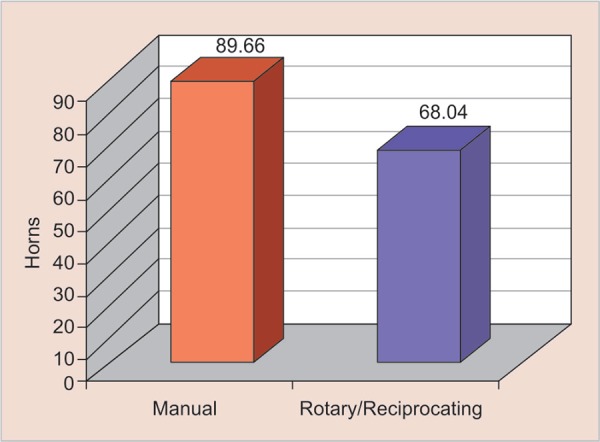
Percentage of time saved between systems (1 month)

**Graph 3:**
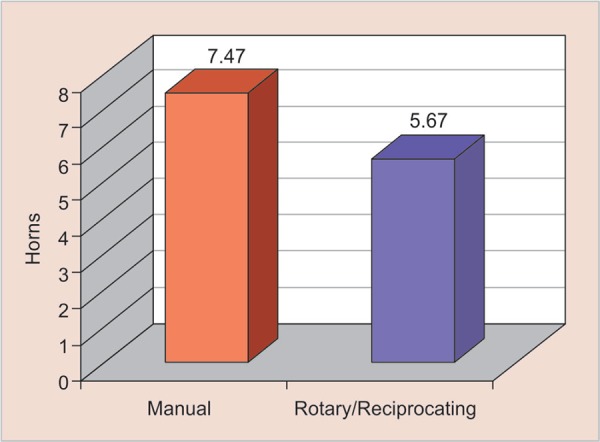
Percentage of time saved between the two systems (12 months)

If we consider these procedures implemented in professional clinical practice for 4 weeks, from the 2nd to the 6th week, and with two patients per day, the rotary and reciprocating systems will have saved approximately 24% of time spent relative to the manual procedure (Graphs 2 and 3).

Considering the practice over the course of one year, at a rate of two patients per day, the time saving is still approximately 24%. Thus, this economical approach could result in 18 more patients being seen per month, as indicated by the formula below.

1.8 hours = 3648 seconds, until 3648/204 = 17.9~18 patients month

## DISCUSSION

Pediatric dentistry's main objective in pulp treatment is maintaining the integrity and health of dental tissues. This is accomplished through the use of techniques and/or medications that allow the continuation of normal development until exfoliation and respect the particular characteristics of the life cycle of these elements.^7^

Comprehensive endodontic therapy for primary teeth can be challenging due to the peculiar anatomy of these teeth, as well as the child's behavior. For this reason, the length of the root must be correctly determined to minimize the apical periodontitis and possible damage to the permanent successor.^3^

Procedures applied in this study demonstrated that modern endodontic techniques had identical effects in reducing treatment time. In addition, this can limit the risk of clinical stress as much as possible.

The rotary technique required significantly less time compared to manual instruments.^4,6,8^ This is consistent with observations from other researchers who have noted that the optimization of endodontic treatment in primary teeth is important because it improves treatment quality and decreases clinical time.^3,6,9–13^ The principles of rotary instrumentation for deciduous teeth are the same as for permanent teeth.

The rotary instruments offer several advantages over traditional stainless steel instruments. They are flexible, have more cutting power, provide better maintenance of the original canal shape, considerably reduce deviation tendency or movement of the foramen, and reduce operative time.^11^

Several studies have been conducted comparing the Ni-Ti instruments’ reciprocating and rotational movement. In evaluating the cyclic fatigue and bending of these instruments, there was a greater resistance observed for files applied in a reciprocal motion relative to the conventional rotational method, longer life-spans for these instruments, and a greater ability to maintain the centralized channel. In addition, the instrument's reciprocating movement caused less movement of apical foramen and less extrusion of dentin debris to the periapex.^14–16^ Results are consistent with the study showing that preparation time with rotary instruments was significantly less than with manual instrumentation, which is a clinically relevant factor for endodontic treatment.

## CONCLUSION

Technological advances have simplified endodontic procedures with regard to the roundabout technique and reciprocation, even in the context of primary dentition, confirming other findings in the literature. However, good treatment also depends on the reduction or elimination of the infectious agent, appropriate instrumentation, efficient irrigation, and shutter-compatible antibacterial materials, as well as knowledge of the case study. However, well-designed follow-up studies for further statistical investigation are necessary for performing endodontic treatment of primary teeth.
